# Ultra rapid lispro improves postprandial glucose control versus lispro in combination with basal insulin: a study based on CGM in type 2 diabetes in China

**DOI:** 10.3389/fendo.2024.1364585

**Published:** 2024-05-07

**Authors:** Lu Yuan, Yi Luo, Yong Luo, Bo Ding, Peng Zhang, Jianhua Ma, Jindan Wu

**Affiliations:** Department of Endocrinology, Nanjing First Hospital, Nanjing Medical University, Nanjing, China

**Keywords:** ultra rapid lispro insulin, postprandial glucose, postprandial glucose excursions, continuous glucose monitoring, HbA1c

## Abstract

**Aim:**

To evaluate the efficacy and safety of URLi (ultra rapid lispro insulin) compared to insulin lispro as bolus insulin with basal insulin using CGM in the individuals with type 2 diabetes(T2D) in China.

**Methods:**

This was a double-blind, randomized, parallel, prospective, phase 3 study. Subjects with uncontrolled T2D were recruited and randomized 1:2 into the insulin lispro and URLi groups. Subjects received a consistent basal insulin regimen during the study and self-administered insulin lispro or URLi before each meal throughout the treatment period. Subjects underwent a 3-day continuous glucose monitoring (CGM) at the baseline and endpoint respectively, and then CGM data were analyzed. The primary endpoint was to compare the difference in postprandial glucose (PPG) control using CGM between the two groups.

**Results:**

A total of 57 subjects with T2D completed the study. Our CGM data showed that postprandial glucose excursions after breakfast (BPPGE) in the URLi group was lower than that in the insulin lispro group (1.59 ± 1.57 mmol/L vs 2.51 ± 1.73 mmol/L, p = 0.046). 1-hour PPG was observed to decrease more in the URLi group than that in the insulin lispro group (-1.37 ± 3.28 mmol/L vs 0.24 ± 2.58 mmol/L, p = 0.047). 2-hour PPG was observed to decrease more in the URLi group than that in the insulin lispro group (-1.12 ± 4.00 mmol/L vs 1.22 ± 2.90 mmol/L, p = 0.021). The mean HbA1c level decreased by 1.1% in the URLi group and 0.99% in the insulin lispro group, with no treatment difference (p = 0.642). In the CGM profile, TBR was not significantly different between the two groups (p = 0.743). The weight gain also did not differ between the two groups (p = 0.303).

**Conclusion:**

URLi can control breakfast PPG better than insulin lispro in adults with T2D in China, while it is non-inferior in improving HbA1c. The incidence of hypoglycemic and weight gain were similar between the two groups.

## 1 Introduction

Diabetes mellitus (DM) has become one of the most common chronic diseases in the world. The global prevalence of diabetes was estimated to be 10.5% in 2021, with China having the largest number of people with diabetes, with more than 140 million, and more than 174 million by 2045 (1). According to statistics, about 95% of Chinese people with diabetes are type 2 diabetes mellitus (T2D) (2). Compared with the Western population, the age of onset of diabetes in Asian patients was generally younger, early β-cell dysfunction was also more obvious in the setting of insulin resistance because there appears to be a predisposition to impaired insulin secretion among East Asian population (3), and polished rice and refined wheat form the basis of most Asian diets with high glycemic index and high glycemic load values (4), postprandial glucose(PPG) fluctuates obviously (5). In the Asian population, postprandial blood glucose levels tend to be higher than in the Caucasian population, even after eating the same foods (6–8). A study conducted in China found that nearly 70% of Chinese T2D patients received insulin therapy, but less than 20% of them reached the glycated hemoglobin A1c (HbA1c) target (HbA1c<7.0) (9).

The HbA1c value is one of the main indicators reflecting long-term glycemic control (10, 11). In order to achieve the HbA1c target value, both fasting plasma glucose(FPG) and PPG should be monitored (12, 13). As HbA1c decreased, PPG had a greater impact on HbA1c than FPG, and PPG accounted approximately 80% of HbA1c when HbA1c was <6.2% and only about 40% when HbA1c was above 9.0% (14). A study in China showed that PPG contributed more than FPG in individuals with HbA1c < 8.5%, whereas FPG became the predominant contributor in the poorly controlled individuals with HbA1c ≥ 8.5% (15). Control of PPG is essential for achieving recommended HbA1c targets. A survey in China showed that the number of participants with isolated fasting hyperglycemia (IFH), isolated postprandial hyperglycemia (IPH) and combined hyperglycemia (CH) were 18.5%, 43.1% and 38.4%, respectively (16). People with diabetes with the IPH phenotype showed increased risks of diabetic microvascular complications compared to participants with the IFH phenotype (16). Clinical studies have demonstrated that targeting PPG can effectively improve glycemic control and long-term results in persons with T2D (17).

However, HbA1c does not necessarily refer daily glucose variability (GV), because the previous studies found that individuals with similar HbA1c may have different GV (18–20). GV is associated with oxidative stress, chronic inflammation and endothelial dysfunction, which contribute to vascular endothelial cell damage (21). Importantly, studies have already demonstrated the positive association between GV and macro/microvascular complications of diabetes (22, 23). Therefore, both HbA1c and GV should be taken into account to reduce the incidence of diabetic complications (20). Continuous glucose monitoring (CGM) continuously provides the glucose readings every 5 minutes for several consecutive days, which may be a potential tool to assess GV in subjects with T2D (24–26).

Reducing postprandial glucose excursions(PPGE), defined as the difference between peak PPG and FPG, is a valuable strategy for reducing GV in the individuals with diabetes (27). Furthermore, the data suggest that PPGE may be a particularly important therapeutic target in person with diabetes. Compared to long-term, sustained hyperglycemia, BG variety postprandially or during glucose ‘swings’ have a more specific triggering effect on oxidative stress, a factor that plays a pivotal role in the development of various diabetic complications (28). There is also evidence that postprandial hyperglycemia is a greater predictor of cardiovascular disease than elevated FPG levels (29).

Besides HbA1c and GV, the scientific community has recently focused on the importance of time in tight range 3.9-10.0 mmol/L (TITR) as a glucose control indicator, correlating with both average glucose levels and GV. TITR is important because it better reflects near-normal, or healthy, glucose physiology than TIR. Low PPGE contributes to achieving tight glycemic control. So the highest TITR may be associated with the lowest PPGE (30–32).

Postprandial glucose can be control with bolus insulin therapy (33, 34). However, the action of many bolus insulins is not sufficiently rapid to match carbohydrate absorption, limiting their efficacy and dosing flexibility (35). Ultra rapid insulins can better match carbohydrate absorption through faster absorption, more rapid onset, and shorter duration of action is highly desired for optimizing PPG control (35).

The active substance of ultra rapid insulin lispro (URLi) is insulin lispro. The excipients contain treprostinil and citrate, which can improve vascular permeability, cause local vasodilation, increase blood flow at the injection site and accelerate the entry of insulin-dependent proline into the vascular circulation to achieve a faster onset of action, shorter duration of action and more effective control of PPG levels (36). Studies have shown that URLi is superior to insulin lispro in controlling PPG levels and has also been shown to be non-inferior in improving HbA1c levels in adults with T2D (37). To date, there has been no study using continuous glucose monitoring systems (CGMS) to evaluate the efficacy and safety of URLi in the treatment of T2D in the Chinese population. Therefore, the aim of this study was to evaluate the efficacy and safety of URLi compared to insulin lispro as bolus insulin (administered 0 to 2 minutes before meal) with basal insulin using CGM in T2D in China.

## 2 Materials and methods

This was a double-blind, randomized, prospective, phase 3 study. The study was conducted in accordance with the ethical standards of institutional and/or national research committees and following the principles of the 1964 Declaration of Helsinki and later amendments. The study protocol and informed consent documents were approved by the Institutional Ethics Committee of Nanjing First Hospital. Written informed consent was obtained from all patients. The trial was registered with ClinicalTrials.gov (NCT03952143).

### 2.1 Participants

From May 2019 to April 2020, T2D individuals in outpatient who presented with poorly controlled blood glucose for at least 90 d were enrolled in the Department of Endocrinology, Nanjing First Hospital, Nanjing Medical University, China. Our site was one of the centers. Data sourced from our center.

The inclusion criteria were as follows: 1) age: above 18 years, 2) T2D duration: at least one year, 3) HbA1c: 7.0% to 11.0% at screening, 4) body mass index (BMI): ≤35.0 kg/m^2^, 5) basal insulin combined with ≥1 prandial insulin or premixed insulin with ≥2 injections daily for ≥90 d prior to screening, 6) combined oral anti-diabetic medication (OAM): no more than three types.

Key exclusion criteria were as follows: 1) any episode of severe hypoglycemia within 6 months prior to screening, 2) one or more episodes of acute complications of diabetes within 6 months prior to screening.

### 2.2 Randomization

Following an eight-week lead-in period, subjects were randomized to receive either URLi or insulin lispro in a 2:1 ratio.

### 2.3 Study design

#### 2.3.1 Insulin titration

The study included a one-week screening period and an 8-week lead-in period, followed by a 26-week treatment period, and a 4-week safety follow-up (**Supplementary Figure S1**). During the 8-week lead-in study period, all the individuals switch from premixed insulin or basal-bolus insulin to basal-bolus insulin. Initial insulin dose allocation: basal insulin accounted for 40-60% of the baseline total daily dose, and meal insulin accounted for another 40-60%. The unit of each meal was assigned by the researchers according to the subjects’ eating patterns. The subjects received a uniform basal insulin regimen during this period: insulin glargine U-100 once daily or insulin degludec U-100 once daily (all the subjects in our site received insulin glargine U-100). The basal insulin dose was titrated according to the median of the last three FBG during the 8-week lead-in period at least once a week, the titration algorithm was in **Supplementary Table S1**. All subjects self-administered insulin lispro before each meal during the lead-in period, and the dose was adjusted under the guidance of the investigator. During the first 12 weeks after randomization (the intensive titration period), the insulin dose at breakfast was adjusted according to the median of the last three self-monitoring of blood glucose (SMBG) before lunch, the insulin dose at lunch was adjusted according to the median of the last three SMBG before dinner and the insulin dose at dinner was adjusted according to the median of the last three SMBG before bedtime at least once a week (**Supplementary Table S2**). During 12 to 26 weeks (the maintenance period), neither prandial nor basal insulin were allowed to be adjusted, except for safety reasons such as hypoglycemia or unacceptable hyperglycemia. From the beginning of the 8-week lead-in period and during the 26-week treatment period, only stable dosing of metformin and/or sodium glucose cotransporter-2 inhibitors (SGLT2is) were continued, and other OAMs were discontinued. The investigator gave the subjects dietary guidance about the meal composition and size.

#### 2.3.2 MMTT

A 4-hour mixed glucose tolerance test (MMTT) was determined for all the subjects at baseline (visit 8) and at the end of the primary treatment period (visit 18), where MMTT at V8 had to be performed before randomization. MMTT required the subjects to be on an empty stomach for at least 8 hours, and patients had to have a FBG range of 3.9-10.0 mmol/L before starting MMTT. The standard meal for MMTT was a liquid nutrient mixture, with individualized insulin doses at mealtime, injected within 0-2 minutes before mealtime, and the subjects completed their meal within 15 minutes, with 0 being the time at which the subject began eating. Venous blood was collected 15 minutes before the meal and 0,15,30,60,120,180 and 240 minutes after the start of the meal (8 times).

#### 2.3.3 CGM

All recruited subjects were subjected to a two-time, 3-day, retrospective CGM (Sof-sensor, CGMS-Gold, Medtronic Incorporated, Northridge, USA) at 3 days before Visits 8 and 18, as described previously (38). During the two-time CGM period, subjects were instructed to maintain moderate physical activity and have breakfast, lunch, and dinner at 07:00, 11:00, and 17:00, respectively, with a total daily caloric intake of 25kcal/kg/day. The percentages of carbohydrates, proteins, and fats were 55%, 17%, and 28%, respectively. After the CGM data collection, glucose indicators, such as the 24hr mean glucose concentration (MBG), 24hr standard deviation of the MG (SD), coefficient of variation (CV), TIR (time in range), TAR (time above range), TBR (time below range), TITR (time in tight range), and postprandial glucose excursions (PPGE) were recorded.

### 2.4 Endpoints

The primary endpoint was to compare the difference of PPGE between the two groups used CGM. The secondary endpoint included HbA1c, other CGM data, hypoglycemia and weight gain.

### 2.5 Statistical analysis

The sample size required was calculated using PASS 15.0. The level of significance, α, was set as 0.05, and the desired power of the study (1 − β) was 90%. Assuming that the mean of PPGE was 2.2 and 2.9 for the URLi and insulin lispro groups, the hypothesized standard deviation (SD) was 0.7 and 0.75 in each group. The minimum number of subjects required was 56 and assuming a 20% drop out rate over 26 weeks. It was estimated that we need enrolled at least 70 subjects.

Data are presented as mean ± SD, median (interquartile range), or percentage as appropriate. Standard t test was used to compare normally distributed data, and the Wilcoxon test was used for asymmetrically distributed data. The categorical data were examined with chi-square test. All statistical analyses were performed using SPSS version 22.0 software (IBM Corp., USA). A p value < 0.05 was considered statistically significant.

## 3 Results

### 3.1 Demographic characteristics

Overall, 75 participants with T2D were assessed for eligibility, 18 participants did not meet the inclusion criteria. Thus, the CGM data of 57 participants were collected and analyzed at the endpoint (insulin lispro, n = 21; URLi, n = 36).

There were no differences in the demographic characteristics of participants between the two groups (**Table 1**).

**Table 1:** The baseline characteristics of subjects of the two groups.

	Insulin lispro	URLi	p value
Gender (M/F)	12/9	20/17	0.820
Age (year)	62.00 ± 6.94	64.70 ± 9.49	0.490
Weight (kg)	65.45 ± 10.40	69.00 ± 9.05	0.180
BMI (kg/m^2^)	24.47 ± 2.38	25.52 ± 2.69	0.144
ALT (U/L)	20.76 ± 9.26	23.68 ± 14.80	0.419
AST (U/L)	19.62 ± 5.27	20.73 ± 9.82	0.633
Cr (mmol/L)	90.40 ± 30.83	78.05 ± 20.89	0.078
HbA1c (%)	8.71 ± 1.00	8.89 ± 1.14	0.549
FPG (mmol/L)	12.05 ± 4.43	12.40 ± 4.52	0.779
Insulin used at study entry (Basal-bolus insulin/Pre-mix insulin)	7/14	12/24	0.611
Time of Pre-mix insulin			0.264
2	12	23	
3	2	1	
Time of Bolus insulin			0.253
2	0	2	
3	7	10	
Insulin dose at study entry (U/d)	42.19 ± 15.07	39.47 ± 15.76	0.522
OAMs used at baseline			
SGLT-2 inhibitors (n)	0	0	/
Metformin (n)	2	8	0.224

ALT, alanine aminotransferase; AST, aspartate aminotransferase; BMI, body mass index; Cr, creatinine; FPG, fasting plasma glucose; HbA1C, Hemoglobin A1c; OAM, oral anti-diabetic medication.

### 3.2 HbA1c

After 26 weeks of treatment, the HbA1c levels in the two groups significantly decreased (**Table 2**). Also in **Table 2**, we showed that there were no differences in the HbA1c levels between the two groups at different stages of treatment. URLi was non-inferior to insulin lispro in terms of the changes in the HbA1c levels from baseline to week 26. The mean HbA1c level decreased by 1.1% in the URLi group and 0.99% in the insulin lispro group with no treatment difference (p = 0.642) (**Table 2**).

**Table 2:** Different stages of treatment of HbA1c in the two groups.

	V1	V8	V18	Δ	p value 1	p value 2
Insulin lispro	8.71 ± 1.00	7.78 ± 0.90	6.80 ± 0.75	-0.99 ± 0.80	0.000	0.000
URLi	8.89 ± 1.14	7.88 ± 1.00	6.77 ± 0.73	-1.10 ± 0.94	0.000	0.000
p value	0.549	0.723	0.913	0.642		

P value 1: V18 vs. V1; P value 2: V18 vs. V8.

Δ: the change of HbA1c from V8 to V18 (V18-V8).

### 3.3 MMTT

The superiority of URLi over insulin lispro in controlling 1-and 2-h PPG was demonstrated during the MMTT. Notably, 1-hour PPG was observed to decrease more in the URLi group than that in the insulin lispro group (-1.37 ± 3.28 mmol/L vs 0.24 ± 2.58mmol/L, p = 0.047). Also 2-hour PPG was observed to decrease more in the URLi group than that in the insulin lispro group (-1.12 ± 4.00 mmol/L vs 1.22 ± 2.90 mmol/L, p = 0.021) (**Table 3**).

**Table 3:** The MMTT profile of the two groups.

Time	Insulin lispro	URLi	p value
Δ-15min	0.22 ± 1.71	-0.12 ± 2.29	0.532
Δ0min	0.37 ± 1.71	-0.16 ± 2.15	0.318
Δ15min	-0.05 ± 2.27	-0.54 ± 2.55	0.463
Δ30min	0.22 ± 3.00	-1.05 ± 2.87	0.128
Δ60min	0.24 ± 2.58	-1.37 ± 3.28	0.047
Δ120min	1.22 ± 2.90	-1.12 ± 4.00	0.021
Δ180min	1.51 ± 2.70	-0.45 ± 4.93	0.060
Δ240min	1.17 ± 3.20	0.04 ± 3.92	0.246

Δ: V18-V8.

### 3.4 CGM profile

Notably, MBG, SD, CV, TIR, TITR and TAR showed no significant differences between the two groups (p = 0.873, 0.582, 0.152, 0.465 and 0.542, respectively) (**Table 4**).

**Table 4:** The CGM profile of the two groups at the endpoint.

Parameter	Insulin lispro	URLi	p value
24 h MBG (mmol/L)	7.80 ± 1.31	7.73 ± 1.54	0.873
SD (mmol/L)	1.90 ± 0.87	2.05 ± 0.92	0.582
CV (%)	22.46 ± 6.71	25.74 ± 9.32	0.152
TIR (%)	84.42 ± 15.86	80.89 ± 17.34	0.465
TITR (%)	69.26 ± 19.72	65.87 ± 22.15	0.581
TAR (%)	0.00 (0.00, 0.18)	0.00 (0.00, 6.51)	0.196
TBR (%)	7.29 (0.00, 19.88)	8.33 (0.00, 17.53)	0.969

CV, coefficient of variation (%); MBG, mean glucose concentration (mmol/L); SD, the standard deviation of the MBG (mmol/L); TAR, time above range (> 10.0 mmol/L) (%); TBR, time below range (< 3.9 mmol/L) (%); TIR, time in range (3.9 - 10 mmol/L) (%); TITR (%): time in tight range (3.9 – 7.8 mmol/L) (%).

PPGE was calculated as the peak value of glucose after meals minus the glucose level at the beginning of each meal. The BPPGE (PPGE of breakfast) in the URLi group was lower than that in the insulin lispro group (1.59 ± 1.57 mmol/L vs 2.51 ± 1.73 mmol/L, p =0.046). The LPPGE (PPGE of lunch) and DPPGE (PPGE of dinner) did not differ between the two groups (p = 0.759 and 0.262, respectively) (**Table 5**). The time to achieve the peak value of glucose after each meal had no difference between the two groups (**Table 5**). TIR and TAR after each meals also showed no significant differences between the two groups (**Table 6**).

**Table 5:** The difference of the PPGE before and after treatment between the two groups.

	Insulin lispro	URLi	p value
BPPGE (mmol/L)			
Baseline	2.99 ± 2.31	3.95 ± 2.93	0.314
Endpoint	2.51 ± 1.73	1.59 ± 1.57	0.046
peak time after breakfast(min)			
Baseline	98.53 ± 52.70	95.54 ± 51.23	0.778
Endpoint	88.23 ± 50.25	77.94 ± 43.87	0.423
LPPGE (mmol/L)			
Baseline	4.07 ± 4.36	3.04 ± 2.42	0.750
Endpoint	2.27 ± 1.81	2.24 ± 1.86	0.759
peak time after lunch(min)			
Baseline	85.67 ± 66.70	65.71 ± 42.60	0.444
Endpoint	64.21 ± 28.25	67.18 ± 40.05	0.824
DPPGE (mmol/L)			
Baseline	3.93 ± 4.30	2.91 ± 2.31	0.731
Endpoint	1.99 ± 1.56	2.60 ± 1.88	0.262
peak time after dinner(min)			
Baseline	87.67 ± 77.04	77.50 ± 48.81	0.888
Endpoint	73.16 ± 55.13	77.21 ± 50.78	0.689

BPPGE, postprandial glucose excursions of breakfast (mmol/L); DPPGE, postprandial glucose excursions of dinner (mmol/L); LPPGE, postprandial glucose excursions of lunch (mmol/L).

**Table 6:** The difference of the postprandial 2h and 4h TIR/TAR before and after treatment between the two groups.

	Insulin lispro	URLi	p value
TIR-2hB (%)			
Baseline	82.02 ± 31.95	73.28 ± 33.76	0.362
Endpoint	83.33 ± 29.22	85.05 ± 23.91	0.821
TIR-2hL (%)			
Baseline	62.50 (20.83, 95.83)	66.67 (9.38, 100.00)	0.873
Endpoint	90.28 ± 20.56	86.76 ± 28.36	0.645
TIR-2hD (%)			
Baseline	70.83 (20.83, 100.00)	66.67 (3.13, 100.00)	0.679
Endpoint	82.41 ± 29.17	89.22 ± 25.89	0.392
TAR-2hB (%)			
Baseline	0.00 (0.00, 33.33)	0.00 (0.00, 42.71)	0.211
Endpoint	0.00 (0.00, 19.79)	0.00 (0.00, 14.58)	0.424
TAR-2hL (%)			
Baseline	16.67 (0.00, 66.67)	33.33 (0.00, 90.63)	0.438
Endpoint	0.00 (0.00, 0.00)	0.00 (0.00, 0.00)	0.793
TAR-2hD (%)			
Baseline	29.17 (0.00, 79.17)	33.33 (0.00, 96.88)	0.808
Endpoint	0.00 (0.00, 39.58)	0.00 (0.00, 0.00)	0.206
TIR-4hB (%)			
Baseline	78.95 ± 28.43	64.15 ± 35.77	0.148
Endpoint	79.51 ± 28.50	83.27 ± 20.67	0.588
TIR-4hL (%)			
Baseline	47.92 (18.75, 89.58)	50.00 (20.83, 90.63)	0.838
Endpoint	85.76 ± 23.29	85.60 ± 28.32	0.983
TIR-4hD (%)			
Baseline	68.75 (14.58, 100.00)	45.83 (8.33, 81.77)	0.524
Endpoint	83.10 ± 25.31	84.99 ± 26.61	0. 806
TAR-4hB (%)			
Baseline	8.33 (0.00, 37.50)	21.88 (0.00, 60.94)	0.150
Endpoint	0.00 (0.00, 43.75)	0.00 (0.00, 25.52)	0.856
TAR-4hL (%)			
Baseline	39.58 (0.00, 81.25)	44.79 (9.38, 79.17)	0.716
Endpoint	0.00 (0.00, 12.50)	0.00 (0.00, 0.00)	0.463
TAR-4hD (%)			
Baseline	31.25 (0.00, 81.25)	46.88 (0.00, 91.67)	0.977
Endpoint	0.00 (0.00, 24.48)	0.00 (0.00, 21.35)	0.338

TAR-2hB: time above range (> 10 mmol/L) from 0 to 120 minutes after the start of the breakfast; TAR-2hD, TAR from 0 to 120 minutes after the start of the dinner; TAR-2hL, TAR from 0 to 120 minutes after the start of the lunch; TAR-4hB, TAR from 0 to 240 minutes after the start of the breakfast; TAR-4hD, TAR from 0 to 240 minutes after the start of the dinner; TAR-4hL, TAR from 0 to 240 minutes after the start of the lunch; TIR-2hB, time in range (3.9 - 10 mmol/L) from 0 to 120 minutes after the start of the breakfast; TIR-2hD, TIR from 0 to 120 minutes after the start of the dinner; TIR-2hL, TIR from 0 to 120 minutes after the start of the lunch; TIR-4hB, TIR from 0 to 240 minutes after the start of the breakfast; TIR-4hD, TIR from 0 to 240 minutes after the start of the dinner; TIR-4hL, TIR from 0 to 240 minutes after the start of the lunch.

**Figure 1:**
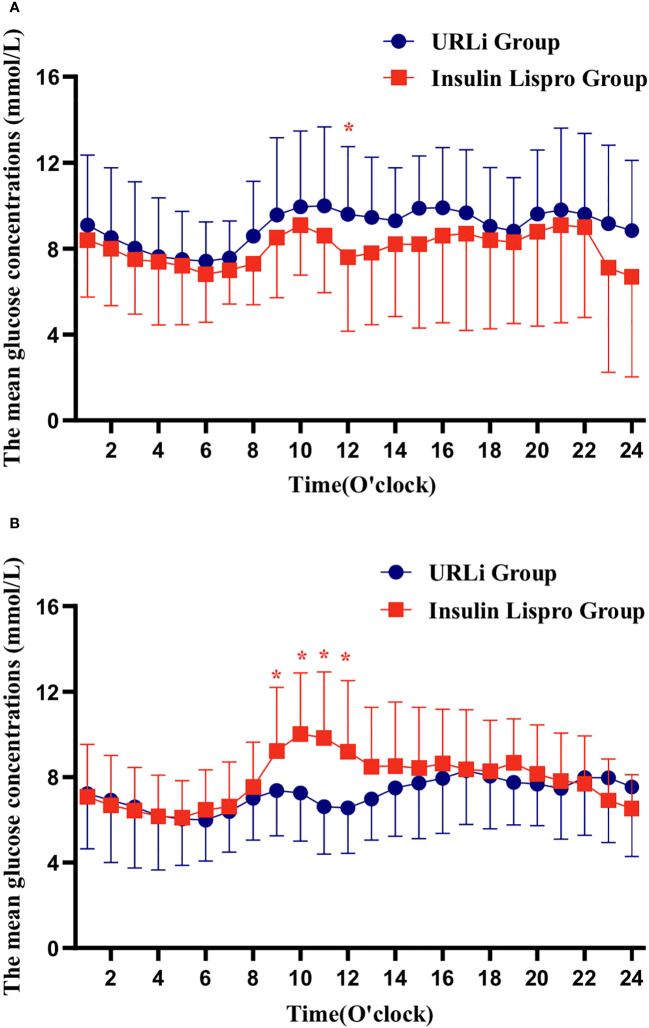
hourly blood glucose concentrations per hour in 24h (**A**: at the baseline; **B**: at the endpoint). *: p < 0.05.

Although the CGM data showed that individuals in the two groups had similar hourly blood glucose concentrations per hour at the baseline, except at 12:00, the hourly MBG concentration at 12:00 in the URLi group was significantly higher than it in the insulin lispro group (**Figure 1A**). At the endpoint, the hourly MBG concentrations at 9:00, 10:00, 11:00, 12:00 in the URLi group were significantly lower than those in the insulin lispro group (**Figure 1B**).

### 3.5 Safety and weight gain

We also compared the risk of severe hypoglycemia (glucose <3.9 mmol/L) between the two groups. Subjects in the URLi group did not show an increased number of hypoglycemic episodes compared with those in the insulin lispro group.

TBR was not significantly different between the two groups (p = 0.743) in the CGM profile (**Table 4**).

The body weight at baseline and endpoint both did not differ between the two groups. The weight gain in the URLi group did not significantly differ from that in the insulin lispro group (2.74 ± 2.36 kg vs 2.95 ± 2.81 kg, p = 0.303).

**Figure 2:**
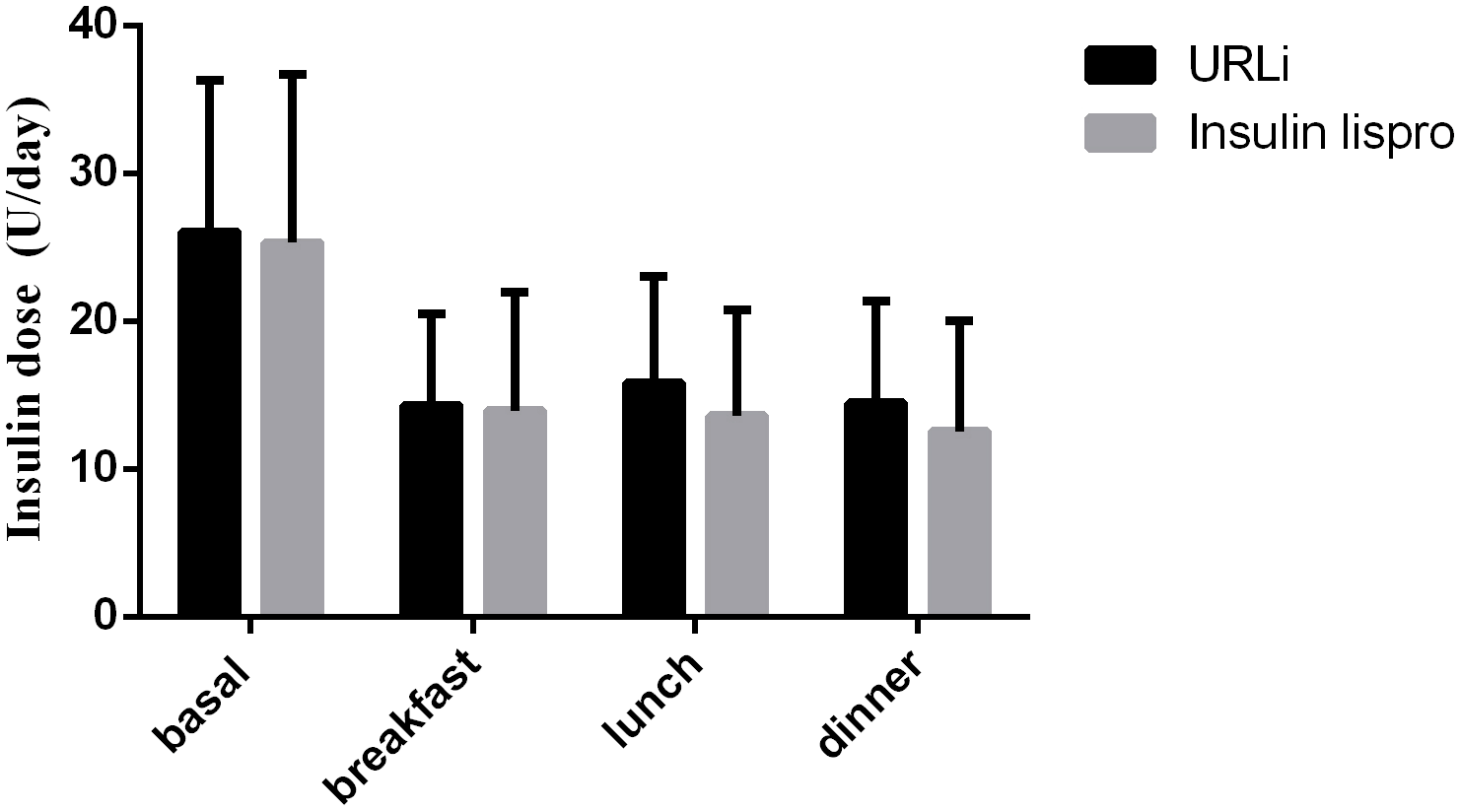
insulin dose between the two groups at the endpoint.

At the endpoint, the basal insulin dose did not differ between the two groups, and the bolus insulin dose also did not differ between the two groups (**Figure 2**).

## 4 Discussion

This prospective study showed that individuals with T2D who received URLi with basal insulin had better postprandial glycemic control than those who received lispro with basal insulin.

Overall, the results of this study suggest that URLi may provide a glycemic control comparable to lispro insulin in individuals with T2D who have significantly elevated PPG. In individuals with T2D, PPG levels typically peak about 2 hours after a meal (39). The increase in PPG is due to loss of insulin secretion in the first phase, decreased insulin sensitivity in peripheral tissues, and decreased suppression of hepatic glucose production after meals (40). Bolus pre-meal insulin treatment reduces PPGE in T2D (41) The first generation of fast acting insulin analogs has shown better PPGE regulation than standard human insulin. However, there is still an unmet need for insulin analogs with faster onset and a shorter duration of action that could potentially contribute to better PPG control than the rapid-acting insulin analogs (42, 43).

Although there was no clinically significant difference in HbA1c reduction between the URLi groups and the lispro insulin group in the study. It is known that HbA1c measurements can be influenced by factors other than glucose levels (44), such as hemoglobinopathies, red blood cell survival, and metabolic factors that influence the glycation response. Information about glycemic variability or the distinction between fasting, preprandial and PPG is not accurately reflected by the HbA1c value (45). It is therefore not surprising that the HbA1c value may not accurately reflect the improvement in average daily blood glucose levels, particularly the reduction in PPGE in individuals treated with URLi. A 1-h plasma glucose cut off of 155 mg/dL post oral glucose tolerance test (OGTT) is an important predictor of developing T2D (46, 47). PPGE is also associated with inflammation, thrombosis, endothelial dysfunction and the development of oxidative stress, all of which may contribute to the pathogenesis of cardiovascular disease (48, 49). Elevated 2-hour PPG levels are associated with an increased risk of cardiovascular events and mortality (50).The MMTT assessment (at breakfast) showed that URLi lowered 1-hour and 2-hour PPG levels and excursions as effectively or in some cases (in the early post-meal phase), even more effectively than pre-meal insulin lispro.

Similarly, CGM profile results at baseline and after 26 weeks of treatment showed that URLi was more effective than insulin lispro in lowering PPG levels and PPGE after breakfast. The effect of URLi was greatest during breakfast. The most important finding of this study is that URLi works particularly well at breakfast, a meal with a high physiological demand for insulin. The BPPGE in the URLi group was lower than it in the insulin lispro group, the URLi can improve the PPGE of breakfast. The peak PPG after breakfast was relatively the highest and reached the peak value the fastest, indicating severe acute postprandial hyperglycemia. In addition to the influence of dietary habits, this was also related to the peak effect of glucose- increasing hormones such as cortisol during this period.

Our results showed a similar safety profile for URLi and insulin lispro. Importantly, the improvement in PPG control with URLi was not associated with an increase in hypoglycemic events.

There was no previous study investigating the efficacy and safety of URLi in the treatment of T2D with CGM. This study has highlighted that URLi can improve PPG more than insulin lispro. One strength of the study is the use of CGM, which can capture more detailed information about blood glucose levels than SMBG and HbA1c, such as PPGE, SD, TIR, TAR and TBR. One limitation of the study is that it did not assess β-cell function in those with T2D. Thus, the result did not account for differences in the efficacy of URLi in individuals with different islet function.

## 5 Conclusion

In conclusion, this study shows that URLi can control breakfast PPG better than insulin lispro in adults with T2D in China, while being non-inferior in improving HbA1c. The incidence of hypoglycemic and weight gain were similar in both groups.

## Data availability statement

The raw data supporting the conclusions of this article will be made available by the authors, without undue reservation.

## Ethics statement

The studies involving humans were approved by the Institutional Ethics Committee of Nanjing First Hospital. The studies were conducted in accordance with the local legislation and institutional requirements. The participants provided their written informed consent to participate in this study.

## Author contributions

LY: Writing – review & editing, Writing – original draft, Investigation, Formal analysis. YiL: Writing – original draft. YoL: Writing – original draft, Investigation. BD: Writing – original draft, Investigation. PZ: Writing – original draft, Investigation. JM: Writing – review & editing, Project administration, Investigation, Funding acquisition. JW: Writing – review & editing, Investigation.
