# Supplementary figures and images for: Ultra rapid lispro improves postprandial glucose control versus lispro in combination with basal insulin: a study based on CGM in type 2 diabetes in China
Source: Front Endocrinol (Lausanne). 2024 May 7;15:1364585. doi: 10.3389/fendo.2024.1364585 (PMC11106447; doi:10.3389/fendo.2024.1364585)

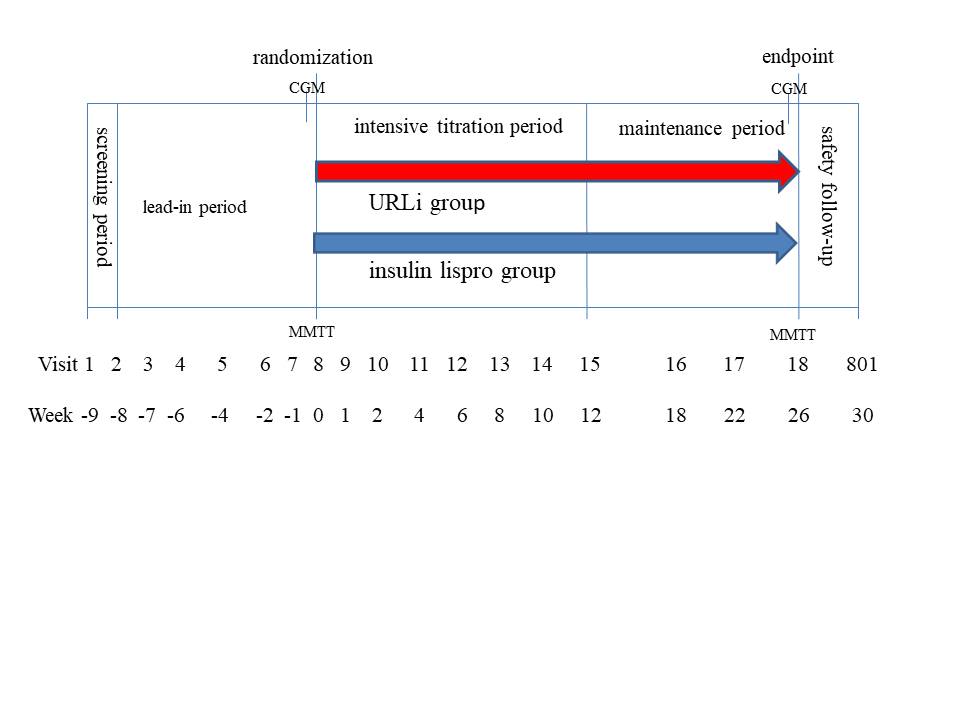

Supplement: Supplementary file 1 [file Image_1.jpeg]
