# Supplementary material for: Ultra rapid lispro improves postprandial glucose control versus lispro in combination with basal insulin: a study based on CGM in type 2 diabetes in China
Source: Front Endocrinol (Lausanne). 2024 May 7;15:1364585. doi: 10.3389/fendo.2024.1364585 (PMC11106447; doi:10.3389/fendo.2024.1364585)
Supplement: Supplementary file 2 [file Table_1.docx]

Table S1 Titration algorithm of basal insulin

| FBG(mmol/L) | Insulin dose (U) |
| --- | --- |
| <4.4 | decrease to the previous lower dose |
| 4.4-5.6 | no change |
| 5.7-7.7 | increase 0 to 2 |
| 7.8-9.9 | increase 4 |
| >=10 | increase 6 |

FBG (fasting blood glucose)

Table S2 Titration algorithm of bolus insulin

| Bolus dose (U) | below the SMBG target | in the SMBG target | above the SMBG target |
| --- | --- | --- | --- |
| <=10 | decrease 1 | no change | increase 1 |
| 11-19 | decrease 1-2 | no change | increase 1- 2 |
| >=20 | decrease 2-3 | no change | increase 2 - 3 |

premeal SMBG target :4.4-6.1 (mmol/L); bedtime SMBG target: 5.0-7.2 (mmol/L)

self-monitoring of blood glucose (SMBG)
